# College Students’ Preferences for Milk Tea: Results from a Choice Experiment

**DOI:** 10.3390/foods12071491

**Published:** 2023-04-01

**Authors:** Xi Lin, Jiangfan Yang, Qian Chen

**Affiliations:** 1 College of Management and Economics, Fujian Agriculture and Forestry University, Fuzhou 350002, China000q25003@fafu.edu.cn (J.Y.); 2 College of Marxism, Fujian Agriculture and Forestry University, Fuzhou 350002, China

**Keywords:** milk tea, tea enterprises, choice experiment, market analysis

## Abstract

(1) Background: Although China is one of the largest tea-producing countries in the world, the Chinese tea industry is facing a decline in profits. However, an explosive market, namely milk tea, has garnered the attention of certain famous tea companies, several of which have launched milk tea products through sub-branding or co-branding. However, there is a scarce amount of literature on consumers’ attitudes toward these marketing strategies of the milk tea market. (2) Methods: Utilizing the choice experiment (CE) approach, the aim of this study was to explore consumer preferences for milk tea and investigate consumers’ socio-demographic characteristics regarding the preference for milk tea. (3) Results: Firstly, although consumers show positive attitudes toward tea bases that come from famous tea companies, they barely pay attention to the types of tea bases of milk tea products. As for ingredients, consumers show significantly negative attitudes toward non-dairy creamers when compared with fruit bases. Moreover, new brands could undermine consumers’ evaluations of milk tea. Secondly, education, the weekly frequency of drinking milk tea, and monthly allowance have a significant influence on consumer preferences. (4) Conclusions: Tea bases from famous tea companies can enhance consumer utility and promote consumer preference for milk tea. Thus, famous tea companies could seek cooperation with milk tea manufacturers, which would be a win–win strategy for both sides. On the other hand, tea companies should make use of their established reputations to gain consumers in the milk tea market, and co-branding or sub-branding strategies could be cost-effective methods to achieve this goal in the highly competitive milk tea market.

## 1. Introduction

Tea is a significant cash crop in China [1]. Not only was the tradition of drinking tea developed in ancient China [2] but China also tops the list among tea-producing countries in the world, ranking higher than India and Kenya [3]. According to the China Statistical Yearbook, complied by National Bureau of Statistics of China, annual tea production in China amounted to 302.6 million tons in 2021 [4]. While tea production is on a steady rise, Chen et al. (2018) mentioned that consumer price index declined in tea purchases, which aligns with the data published in the China Statistical Yearbooks from 2017 to 2021. However, the producer price index for tea simultaneously kept growing [4,5]. In other words, this phenomenon indicates a higher cost of tea production and a lower price of tea in the markets. Hei et al. (2022) pointed out a decline in the percentage of earnings from tea over CNY 10,000 [6]. As the second largest producer of tea, India is also facing a similar challenge as half of their tea produce is sold below the cost of production [7]. Moreover, the outbreak of COVID-19 pandemic has adversely affected the global markets and changed people’s consumption trends, resulting in consumers spending more on daily necessities and medicines rather than certain non-essential goods, such as tea [6,8,9]. Meanwhile, some studies have opined that the current Chinese tea industry has failed to attract the group that is rapidly increasing in size and purchasing power in tea and beverage markets [5,6,10], namely, individuals born between 1996 and 2012, also known as Generation Z (Gen Z) [11,12,13]. According to Tanui et al. (2012), it is crucial for tea companies to diversify products in other market segments to attract new consumers [14]. One promising strategy is to develop a new tea-based market beyond traditional method of making tea by soaking tea leaves in boiling water [15].

A recent study on the beverage market has found that milk tea is a popular product most frequently mentioned by consumers [16]. Milk tea is a tea-based beverage invented in the Taiwan Province in 1984 [17]. Since then, the milk tea business has been growing rapidly in China [13]. It was estimated that the milk tea market amounted to more than CNY 140.5 billion in sales in 2019, highlighting a significant expansion [13]. The China Chain Store and Franchise Association issued a report on the milk tea market in 2022, naming milk tea as a new type of beverage, in order to separate this new tea-based beverage from the traditional form of tea. This report highlights that the explosive milk tea market helped to increase the sales volume of tea [18]. The key to improving profits for tea companies is to develop products that are able to yield a high-added value [19,20]. The rapidly expanding milk tea market presents tea enterprises with many new opportunities, rather than solely needing to depend on the sale of tea leaves [18]. In the beer industry, co-branding can be a cost-effective marketing strategy in a highly competitive mature industry [21]. A recent report points out that several famous tea companies have launched their milk tea products via sub-branding or co-branding. For example, Nayuki, a brand founded in 2015, which now ranks second in the Chinese milk tea market with a market share of 18.9%, created a co-branding product that combines tea with milk or fruit in July 2022. Another famous tea enterprise, Chunlun, established a subsidiary milk tea brand to attract young consumers [22]. As these strategies are still in their infancy, the question remains as to whether these marketing strategies benefit tea companies in the highly competitive milk tea market.

Previous studies on milk tea have mainly concentrated on two aspects. The first is related to the physiological aspects of milk tea: for example, how nutrients in milk tea are absorbed by human bodies [23,24]; whether milk tea poses health risks, such as obesity, due to its sweetness [25,26]; or questions concerning caffeine intake from milk tea [27]. The second is focused on the factors that can influence consumers’ purchase decisions when they buy milk tea. A study conducted in 2019 mentioned that Chinese consumers’ purchase decisions were influenced by price, the pursuit of a pleasant state of mind, and novel experiences [28]. Van Dung HA (2020) used exploratory factor analysis (EFA) and the multivariate regression model to analyze the seven determinants of milk tea selection, whose results showed that shop location had the most significant impact on milk tea selection, while customer service had the smallest impact [29]. De Guzman et al.’s (2020) study of the influence of marketing strategies of milk tea shops also drew a similar conclusion wherein they found that consumers pay more attention to the location of milk tea shops and price [30]. Lee et al. (2021) also mentioned that milk tea price-setting is the most significant factor affecting consumers’ psychological purchase decisions [31]. Ong et al. (2021) studied consumer preference for milk tea through a conjoint analysis approach and found that the internal attributes of milk tea, such as pearl size, sugar level, the amount of ice, the type of drink, and the price, influenced consumer preferences [32]. Another study by Istijanto et al. (2021) found taste, texture, packing, store, price, health, trend and brand playing important roles in explaining the purchasing behaviors of Gen Z consumers [33]. It can be concluded from above that attributes of milk tea can influence consumers’ preferences regarding milk tea. However, Gao et al. (2019) also pointed out that the attributes of products are not the only features to exert influence on consumer preferences; they are also influenced by the socio-demographic characteristics of the consumers [34].

Of all the internal attributes of milk tea, the tea base itself was usually ignored by consumers. However, with the diversification of milk tea products and tea companies’ co-branding strategies, the extrinsic characteristics, such as the type of tea or tea enterprises’ brands, as shown on menus, cause consumers to pay much more attention to the tea-based aspects [22]. Nevertheless, there is little research on these factors. Lin et al. (2019) argued that in order to prevent milk tea companies from substituting domestic high-priced teas with low-priced imported teas, the Food and Drug Administration of Taiwan Province issued a bulletin in 2016 stating that the origin of the tea leaves should appear on the packaging of beverages [35]. This means that tea leaves themselves represent a part of the reputation of milk tea products. However, it is rare for the literature to discuss how this impacts consumers’ milk tea preferences, although some studies have proven that brand awareness can influence consumers’ purchasing intentions [36,37,38]. A study on milk tea in Malaysia revealed that brand awareness had a significant positive impact on brand loyalty, which can be applied to gain a competitive advantage [39]. However, whether the reputations of famous tea companies are relevant in the milk tea industry is open to debate. 

The choice experiment (CE) method, an approach involving stated preferences used to determine consumer preferences for certain products or services, has been adopted in many recent studies on beverage products [33,40,41,42]. Consumer intention is driven by multiple attributes, and the CE method is effective in measuring consumer preferences for each attribute and interaction effect between various factors [34]. Caputo et al. (2022) states that CE can be used to analyze consumers’ demands for novel products [43]. Even though milk tea beverages have started providing information concerning tea leaf origin or tea companies, no studies have examined how this information affects consumer preferences for the tea-based aspect of milk tea. Therefore, this study applies the CE method to precisely analyze consumer milk tea preferences.

All in all, the milk tea market is still developing rapidly and is highly competitive, which makes it crucial for tea companies to understand how to implement new and effective strategies to engage in the milk tea industry so as to reverse their falling profits from traditional sales of tea. This study attempts to explore consumer milk tea preferences through a CE method in order to help tea enterprises implement effective marketing strategies to improve profits. In other words, the aim of this study is two-fold. Firstly, it aims to determine the factors affecting consumer milk tea preferences, ranging from attributes such as the origin of the tea leaves, types of tea base, and added ingredients to the milk tea brands and the price. Secondly, it discusses how consumers’ socio-demographic characteristics impact their milk tea preferences. The results of this study could be useful for tea enterprises to understand young consumers and could help them make their marketing strategies more effective.

## 2. Experimental Procedures and Data

### 2.1. Attribute Selection

Fujian plays a significant role in the Chinese tea industry, as the production of tea in Fujian is the highest in all of China [4], and many famous tea brands have been established in this province [44]. On the other hand, most people who live in Fujian are in the habit of drinking tea, and are thus more sensitive to the extrinsic aspects of tea, which is conducive to measuring consumers’ preferences more precisely. Thus, this research was conducted in Fuzhou city, the provincial capital of Fujian Province. 

The attributes examined in this study were obtained from real market research and previous studies. When conducting market research on milk tea, our research team recorded and analyzed the menus of famous milk tea shops in the Fujian Province, such as CoCo, Gongcha, Alittle, Nayuki, and Heytea, which are the most popular brands across China [22,32,45]. As milk tea consist of a tea base and other ingredients [46,47], attributes were chosen based on those two categories and comprised the type of the tea base, the origin of tea leaves, added ingredients, and the milk brand. Based on previous studies, price appeared to be an important factor, so it was also studied in this research. All the attributes are shown in Table 1.

**Figure 1:**
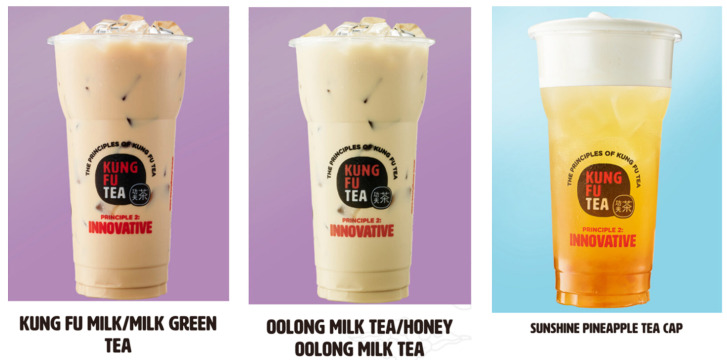
Examples of milk tea beverage menu in the realistic market.

Tea is generally classified into three groups based on fermentation level, namely black tea, green tea, and oolong tea [48]. Studies on milk with green or black tea date back to 1998 [49], but most of these were studies concerning the biochemical changes that occurred in green or black teas with milk [49,50]. How consumers evaluated the different types of tea bases in milk tea has only rarely been discussed in previous studies. This study provides an example of milk tea products in the actual milk tea market, and we found that menus do not provide information about the types of tea base on all milk tea products (Figure 1). In line with the real-world market, we set “none” as a category for the “type of tea base” attribute in order to imitate real milk tea beverages without tea base information. Moreover, jasmine tea is a unique reprocessed tea in Fuzhou, China, where jasmine has been grown in large quantities since ancient times [51]. We added jasmine as a tea base choice, since it can be easily found on the Fuzhou milk tea market.

According to the literature, product quality depends on the quality investment of the manufacturer and the supplier [52]. A famous brand is a symbol of high quality, which may have a positive effect on consumers’ purchase decision [53]. Consumers’ preferences and willingness to pay (WTP) for products are not only affected by the quality but also by the product’s brand. Moreover, the brand image of a product is not only affected by the producer’s image but also by the image of related suppliers, a phenomenon identified as the “brand halo” effect by Xu et al. (2019) [52]. Liu et al. (2019) confirmed that tea from famous tea companies is regarded as a guarantee of quality [54]. As the raw material provider, it is necessary to explore whether the “brand halo” effect of tea companies exerts any influence on milk tea beverages. Thus, we set two categories of tea leaves: tea leaves provided by famous tea enterprises or “none”, i.e., no information.

Today, young consumers are adventurous and tend to try new tastes [55]. Most milk tea recipes contain a tea base and milk, although other ingredients, such as fruit, are also found in the current Chinese milk tea market [22,56]. Moreover, regular milk tea is made up of a tea base and fresh milk or non-dairy creamer [32]. Non-dairy creamer may suit some consumers with digestive issues, such as lactose intolerance [57], but health problems caused by non-dairy creamer have impacted consumers’ purchasing decisions to such an extent that milk tea manufactures have opted to use fresh milk in certain products [58].

As for the various brands of milk tea products, the future milk tea market is predicted to bloom and continue to be competitive, and as such, there are various directions in which brands can develop, such as implementing personalized branding [45]. Taking the marketing strategies of realistic milk tea market into consideration, current brand subsidiaries of famous tea brands and new brands are chosen as the different levels of attribute. These branding tactics are shown in Table 1 and are studied in this paper.

As the majority of milk tea consumers are Gen Z, this means that they are much more price conscious [33,59]. Price is one of the decisive factors that affect consumers’ purchasing intentions [60]. To encourage or to curb the consumption of certain types of products, effective subsides or taxes range from 10% to 20% [61]. Since the retail prices of milk tea are cheap in the real-world market, we set a 20% rate to ensure that price changes could be identified by respondents. In line with the real-world market, price was rounded up to the nearest integer.

### 2.2. Experimental Design

**Figure 2:**
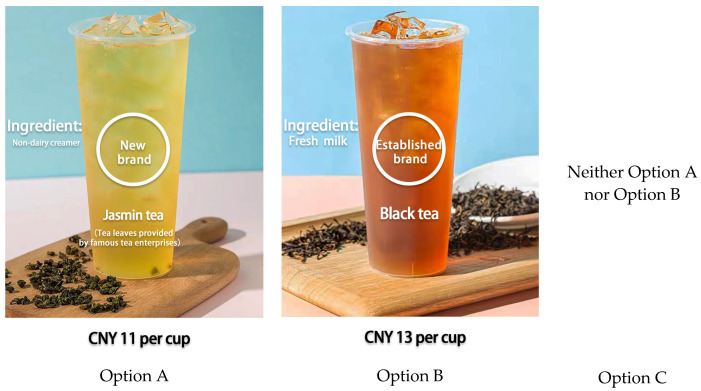
An example of a choice set in the questionnaire.

The structure of the choice sets is vital to the accuracy of the CE method; therefore, the designed choice sets should explain the variance of attributes to a significant extent while minimizing random errors [62,63]. This study adopted an opt-out option designed to imitate consumers’ purchase decisions in a realistic market, which can be seen in studies using a non-hypothetical CE study. The opt-out option can make the research more accurate [64,65]. In total, three options were provided for each selection (Figure 2). We designed a virtual menu of milk tea beverages, tea base types were printed on the cups of milk tea, and consumers were required to make a choice based on the provided ingredients and other related information.

A full factorial design for choice sets amounts to a total of 216 (4 × 2 × 3 × 3 × 3) factors, and choice sets grow exponentially for every two alternatives that are involved in one selection. However, it is impossible to conduct a full factorial design research due to limited funds and resources. On the other hand, a fractional factorial design is an improved method that maintains the efficiency of research but reduces the number of choice sets [66]. Therefore, a D-optimal factorial design was adopted in this study and thirty-six choice sets were generated, of which the D-efficiency and D-error were 97.16% and 0.06, respectively. These thirty-six choice sets were randomly divided into nine blocks, and each block had four choice scenarios. One version of the questionnaire consisted of a block of choice sets, socio-demographic information, and subjective knowledge of milk tea. Each respondent answered one of the nine versions of the questionnaire.

### 2.3. Data Collection 

Many studies stated that the main consumers of milk tea are the so-called Gen Z, defined as individuals born between 1996 and 2012 [46,67], who are mostly students at present. In this study, college students were chosen as the representative samples for two reasons. First, compared with the limited allowance of students in high school, most Chinese college students receive a monthly allowance from their parents, and they are able to make their own purchasing decisions. Taking this into account allows us to investigate consumer preferences more accurately. Second, the CE method involves multiple choices and college students have a better understanding of these complicated questionnaires.

We conducted a face-to-face questionnaire survey in nine public universities located in Fuzhou from November to December 2022. Fifty respondents were randomly chosen in each university. Nine questionnaire version of this study were provided online, and respondents were asked to fill in the questionnaire, which was accessed by scanning a QR code. 

Finally, we invited a total of 450 students to participate in our research. We set a “trap question” in the questionnaires, according to the suggestion by Wang et al. (2017). This method was used to identify careless respondents [68]. The “trap question” was set as the following question: “Please select the ‘yellow’ option in the following four options.” The questionnaire was not considered if any other color option was selected. Ultimately, we obtained a total number of 432 valid questionnaires (96.00%). All data were calculated using Stata 15.0.

### 2.4. Models 

The Lancaster consumer theory states that consumer utility is derived from the attributes of the product rather than the product itself [69]. Thus, consumer utility consists of two parts, namely the observable representative and unobservable random error terms [70]. This can be expressed in a mathematical equation as follows:

(1) $$U_{nit}=V_{nit}\left(\beta_{n}\right)+\varepsilon_{nit}=\delta \left(ASC\right)+a_{n}\left(X_{i}\right)+\gamma_{n}\left(-P_{i}\right)+\varepsilon_{nit}$$

where *U_nit_* is the utility of individual *n* from the given alternative *i* of the choice set *t*; *V_nit_(β_n_)* is the observable utility with parameter *β_n_*; and *ε_nit_* represents the random error. The observable utility derives from the attributes of the given alternative *i*, which is expressed as the vector *X_i_*. *P_i_* is the expenditure on the alternative *i*. *ASC* reflects the “none” option, where the value is 0, and the value being 1 indicates the selection of the designed choice sets.

Among the logit models, the formation is determined through the hypothesis of the random error distribution and heterogeneity [71]. The multinomial logit model is the basic form of logit modeling, which assumes that the consumers have a homogeneous preference for the product [40]. However, Gao et al. (2019) pointed out that consumers share inconsistent preferences for the product in realistic markets, and the random parameter logit (RPL) model can relax the hypothesis of consumers, which is consistent with reality [34]. Hence, the RPL model was adopted in this study to evaluate consumer preferences for milk tea.

The main effects of the attributes in this study can be expressed by Equation (2). Consumer utility is derived from the five chosen attributes in Table 1, “Tea”, “Types”, “Ingredients” and “Brand” are the abbreviations of “Tea leaf origin”, “Type of tea base”, “Added ingredients” and “Milk tea brand”, respectively (Table 1). Individual *n* chooses the alternative *i* in the choice set *n*, which is expressed as *nit*. Additionally, the parameter vectors *β*_1_ to *β*_5_*_n_* represent the degrees of the individual *n*’s preferences for the relative attributes that are necessary to determine. The “Price” attribute is the metric variable, while the other four attributes are nominal variables. “Types” and “Tea” attributes were coded with the baseline category “none”, and the “fruit” and “established brand” categories were used as the baselines of the “Brand” and “Ingredients” dummy variables.

(2) $$U_{nit}=ASC+\beta_{1}Price_{nit}+\beta_{2n}Types_{nit}+\beta_{3n}Tea_{nit}+\beta_{4n}Ingredients_{nit}+\beta_{5n}Brand_{nit}+\varepsilon_{nit}$$

Individual *n*’s WTP for attribute x is estimated as Equation (3):

(3) $$WTP_{n}=-\frac{\beta_{nx}}{\beta_{np}}$$

where *β_nx_* represents the coefficient of the non-price attribute *x* and *β_np_* is the coefficient of the price attribute *np*.

## 3. Results

Of the respondents, around 61.34% were female, which is in line with many studies, where female respondents slightly exceed male respondents [16,30,32]. Over 87% of participants were junior college students or undergraduates, while postgraduates comprised 12.27%. As for the weekly frequency of drinking milk tea, only 39 (9.03%) respondents drank milk tea more than four times a week, while most participants consumed milk tea no more than once per week. This result is analogous to the study by Ong et al. (2021), which found that most consumers drink milk tea less than three times a week, and those who drink milk tea once a week account for the largest proportion [32]. In this study, the target demographic was students, around 50% of whom were provided with a monthly allowance between CNY 500 and 1499 and 31.94%received an allowance between CNY 1500 and 2499, while only 14 people had a monthly allowance of more than CNY 3500 (Table 2).

### 3.1. Main Effects of the Attributes

The results of main effects of the attributes are shown in Table 3. Firstly, the coefficient of price is statistically significant at the 1% level, which implies that participants show negative attitudes towards price increases. We found that when compared with no information, tea leaves supplied by famous tea enterprises enhanced the utility of consumers. As to the types of tea bases, consumer preferences did not differ, which indicates that consumers pay little attention to the types of tea bases in milk tea products. On the other hand, the coefficient of non-dairy creamer is negative, which means that compared with fruit, consumers show significantly negative attitudes toward non-dairy creamer; however, there is no difference between fruit and fresh milk. Furthermore, the coefficient of a new brand is significantly negative at the 5% level, which indicates that a milk tea product of a new brand would reduce the utility of consumers—this also suggests that it is hard for new brands to attract consumers in the milk tea market. All in all, the results correspond to the proposition stated in the introduction that it would be beneficial for famous tea enterprises to provide tea leaves for the milk tea industry, and this could be an effective extrinsic characteristic regarding consumer milk tea preferences.

### 3.2. Main Effects of Socio-Demographic Interactions

That consumers have heterogeneous preferences for different products has been confirmed by many studies [34,40]; thus, this section specifically determined the interaction effects of attributes and the socio-demographic characteristics of participants. Results are shown in Table 4. Education, the weekly frequency of drinking milk tea, and monthly allowances all have a significant influence on consumer preferences. As seen in Table 4, individuals with a higher education level display negative attitudes towards tea leaves provided by famous tea companies. On the other hand, well-educated participants were inclined to choose milk tea from subsidiaries of famous tea brands. Furthermore, these attributes enhanced consumers’ utility if they drank milk tea multiple times a week, thus indicating that the milk tea industry should continue to improve the attributes of milk tea so as to maintain their existing consumers. With regards to the influence of monthly allowance on the purchase of milk tea, students with higher monthly allowance preferred milk tea made from tea leaves provided by famous enterprises, but a negative value was found for black and green tea bases. The results also show a significant negative value for non-dairy creamer, indicating that milk tea manufacturers should avoid using non-dairy creamer when focusing on mid-range products [45].

### 3.3. Consumer WTP in Terms of Attributes

Consumer WTP values for given attributes were also calculated and are shown in Table 5. It can be seen that milk tea with non-dairy creamer has a strong negative value for consumer milk tea preferences, which has the highest premium of CNY 10.40. Similarly, young consumers also show negative attitudes towards milk tea from new brands, involving a CNY 1.43 premium for the attribute. On the other hand, consumers prefer tea leaves provided by famous tea enterprises, with the WTP values for the product at about CNY 1.12. The results indicate that milk tea products without non-dairy creamer and from established brands or their subsidiaries may have a positive influence on consumer preferences, and that tea leaves provided by famous tea companies lead to higher WTP values for the milk tea products.

## 4. Discussion

Seeking new markets is of great significance for Chinese tea companies in pursuit of profit growth [14,15]. The younger generations are the main consumers in the current domestic beverage market and they are increasingly purchasing milk tea [18]. As such, some tea enterprises have joined the milk tea industry in an attempt to attract these young consumers [5,18]. Therefore, it is essential to understand the consumer preferences of this young demographic regarding milk tea. The previous literature focused predominantly on the internal attributes of milk tea, such as sugar level, the amount of ice, pearl size, and so on [32]. However, this study placed emphasis on the tea-base part of milk tea and undertook an in-depth examination of the influence of tea base types, and in particular, the origins of tea leaves on consumer’s milk tea preferences. Ingredients and brands of milk tea were also analyzed in this study to measure the utility of consumers.

In this study, respondents showed positive attitudes towards tea bases from famous tea enterprises, which indicated that consumer utility enhanced through the use of tea leaves from famous tea companies, and consumers also showed a willingness to pay a higher price for such milk tea products. This result is in line with the study by Xu et al. (2019), who stated that the supplier also has an effect on the brand image of the final product, called the “brand halo” effect [52]. This is because tea leaves are an important ingredient in milk tea, and when they originate from established tea companies, it can promote milk tea products’ brand images and encourage purchasing behaviors. On the other hand, many studies are concerned about functions or nutritional value of different types of tea [35,48]. Several studies have examined the chemical functions and the taste of milk tea products made with different types of tea [72,73]. Another study explored the color and taste of milk tea made with large-leaf yellow tea [74]. In the traditional tea market, the consumption of tea varies by tea type. [4]. However, in this study, participants rarely paid attention to what type of tea is used to make the tea base when considering the purchase of milk tea. This result was different from the consumption behaviors in the traditional tea market [75], which implies that marketing strategies should focus on attributes other than types of tea in order to attract younger consumers in the milk tea market. 

Regarding the brand of the milk tea products, the results of this study show that participants were reluctant to buy milk tea products from new brands. This result is similar to the study by Choi et al. (2019), who found that consumers were willing to pay a higher price for a well-known global brand [16]. Similarly, a study conducted in the Malaysian milk tea market found that brands had a significant effect on consumers’ purchasing intentions for milk tea [76]. According to Jin et al. (2013), a brand serves as a way to build consumers’ recognition of certain products [77]. The potential reason for the result is that consumers tend to undermine the quality of a new brand [78]. Thus, it is not easy to enter the competitive milk tea market by building a new brand. 

Many ingredients added into milk tea can be found in the current Chinese milk tea market [22]. We chose the most popular ingredients in this study, namely fresh milk, fruit, and non-dairy creamer, and our results show that consumers’ evaluation of non-dairy creamer is negative. A possible reason for this may be the food scandals related to non-dairy creamer in China which have caused health concerns among consumers [58]. Aside from this, there is no statistical significance between fresh milk and fruit in this study; however, this result is the opposite of the results of Ong et al. (2021), who found that people preferred fresh milk to fruit in milk tea [32]. These inconsistent findings are considered to be a consequence of different eating habits in different countries. In conclusion, manufacturers should use fresh milk or fruit instead of non-dairy creamer to encourage consumers’ purchasing intentions.

Taking the socio-demographics of participants into consideration, we found that consumers with higher levels of income are more inclined to choose milk tea with a tea base made from tea leaves provided by famous enterprises, but they are reluctant to purchase milk tea that provides information about the type of tea used. According to Wang et al. (2019), one of the factors that influences Chinese consumers’ purchasing intentions when buying tea drinks is that they tend to pursue novel experiences [28]. This indicates that young consumers seem to show negative attitudes towards traditional attributes of a new product. Moreover, consumers with higher education levels tend to purchase milk tea from subsidiaries of famous tea brands, and this indicates that famous tea enterprises should use sub-branding strategies to expand into the new milk tea market. As for other socio-demographic variables, no significant interactions with milk tea were found in other studies, and hence they are not included in this study.

### 4.1. Practical Applications

Many studies have suggested that tea enterprises should start pilot development projects in order to revive the tea industry [4,5,6]. However, few talk about what projects are suitable for tea companies. The boom in the milk tea market has attracted several tea enterprises. According to results of this study, tea bases from famous tea companies can enhance consumers’ utility and promote consumer preference for milk tea products. Thus, famous tea enterprises could seek cooperation with milk tea manufacturers, which is a win–win strategy for both parties. However, there are many players in the milk tea market who provide consumers with far more choices than they need, which means that new brands do not have many good opportunities to enter the milk tea market. To be specific, tea companies should make use of their established reputations to attract consumers in the milk tea market, and co-branding or sub-branding strategies are the cost-effective methods that can be employed to achieve this goal. On the other hand, our study found that consumers rarely pay attention to the types of tea in the milk tea products. Similarly, Yang et al. (2011) mentioned that the Chinese tea industry is facing a dilemma, in that they have famous tea but do not have famous brands [79]. This implies that the marketing strategies of tea companies should be focused on brand establishment rather than developing famous types of tea. Moreover, since customers give more importance to the ingredients of milk tea due to concerns about non-dairy creamer, marketers can highlight the natural ingredients (i.e., fresh milk and fruits) to attract more consumers.

### 4.2. Study Limitations

The limitations and future research directions of this study are as follows. Firstly, this study places more emphasis on the attributes that are relative to the aspects of tea. However, as a type of novel drink, milk tea is now undergoing rapid changes in terms of taste, packing, texture, and so on, which also play important roles in explaining consumer’s milk tea preferences. These were not explored in this research. Although types of tea base seem to have no influence on consumers’ purchasing intentions, which we attempted to explain through their pursuit of novel experiences, in future research, it will still be necessary to analyze why young consumers pay no attention to tea types when they purchase milk tea, especially in a country that has a long history of drinking tea and a highly developed tea culture.

Secondly, a study on the current brands in Chinese milk tea market found that brands should target consumers according to their income level [45]. Although we discussed the impacts of socio-demographic characteristics on consumer preferences for milk tea in this study, it would be worthwhile to delve into marketing strategies that are more relevant to the target consumers and brand positioning.

## 5. Conclusions

This study demonstrates that tea bases made with tea leaves provided by famous tea enterprises enhance consumer utility when compared with those made with tea leaves from unknown suppliers. Moreover, the “non-dairy creamer” coefficient is negative, which means that compared with fruit, consumers show significant negative attitudes to non-dairy creamer and are reluctant to choose milk tea with non-dairy creamer. Furthermore, the “new brand” coefficient is significantly negative at the 5% level, which indicates that a milk tea product of a new brand reduces the utility of consumers. It is important for tea enterprises to establish a reputation before entering the milk tea market. In general, it is beneficial for famous tea enterprises to provide tea leaves for the milk tea industry, as this could be an effective extrinsic factor in consumer’s milk tea preferences.

Moreover, socio-demographic characteristics also influence milk tea purchase. Education, weekly frequency of drinking milk tea, and monthly income all have a significant influence on consumer preferences. Our results found that people with higher education levels are inclined to choose milk tea from subsidiaries of famous tea brands. Regular consumers display a higher opinion of milk tea products when attributes are provided. Additionally, students with higher monthly allowances prefer milk tea made with tea leaves provided by famous companies, but a negative value was found for black and green tea bases. This study also shows significant negative values for non-dairy creamer.

## Figures and Tables

**Table 1:** Product attributes and levels in the choice experiment.

Attributes	Number of Levels	Levels
Type of tea base	4	None Black tea Green tea Jasmine tea
Tea leaf origin	2	None Provided by famous tea company
Added ingredients	3	Fruit Fresh milk Non-dairy creamer
Milk tea brand	3	Established brand Subsidiary of famous tea brand New brand
Price	3	CNY 11 per cup CNY 13 per cup CNY 16 per cup

**Table 2:** Socio-demographic and economic characteristics of participants.

Variable	Definitions	Frequency (N = 432)	Percentage
Gender	Male	167	38.66%
	Female	265	61.34%
Education	Junior college student	176	40.74%
	Undergraduate	203	46.99%
	Postgraduate	53	12.27%
Frequency of weekly drinking milk tea	0–1	247	57.18%
	2–3	146	33.80%
	4–5	27	6.25%
	≥6	12	2.78%
Monthly allowance	≤500	59	13.66%
(CNY)	500–1499	201	46.53%
	1500–2499	138	31.94%
	2500–3499	20	4.63%
	3500–4499	1	0.23%
	4500–5499	3	0.69%
	≥5000	10	2.31%

**Table 3:** Estimation of the main effects of attributes using the RPL model.

	Variables	Coefficients	Std. Error	*p*
Random parameter means	ASC	−5.133	0.622	0.000
	Price	−0.200	0.034	0.000
	Provided by famous tea enterprises	0.225	0.102	0.028
	Black tea	0.089	0.174	0.609
	Green tea	−0.053	0.186	0.777
	Jasmine tea	0.083	0.150	0.580
	Fresh milk	0.094	0.143	0.509
	Non-dairy creamer	−2.081	0.329	0.000
	Subsidiary of famous tea brand	0.216	0.142	0.128
	New brand	−0.286	0.135	0.033
Random parameter distribution	ASC	4.652	0.483	0.000
	Provided by famous tea enterprises	0.366	0.311	0.239
	Black tea	1.276	0.346	0.000
	Green tea	−0.055	0.427	0.897
	Jasmine tea	0.024	0.420	0.955
	Fresh milk	1.698	0.288	0.000
	Non-dairy creamer	2.355	0.394	0.000
	Subsidiary of famous tea brand	0.695	0.344	0.043
	New brand	−0.532	0.394	0.177
Log-likelihood		−1411.738		
LR chi2 (7)		657.910		

**Table 4:** Estimation of the interaction effects of attributes with socio-demographic variables using RPL models.

	Variables	Coefficients	Std. Error	*p*
Random parameter means	ASC	0.698	1.412	0.621
	Price	−0.188	0.036	0.000
	Provided by famous tea enterprises	0.292	0.500	0.559
	Black tea	0.235	0.989	0.813
	Green tea	−0.021	0.914	0.981
	Jasmine tea	−0.374	0.715	0.601
	Fresh milk	−0.295	0.723	0.683
	Non-dairy creamer	0.076	0.825	0.927
	Subsidiary of famous tea brand	0.347	0.730	0.634
	New brand	−0.206	0.651	0.752
	gender × ASC	−0.812	0.674	0.228
	gender × provided by famous tea enterprises	0.184	0.253	0.467
	gender × black tea	0.152	0.430	0.723
	gender × green tea	0.018	0.442	0.967
	gender × jasmine tea	0.459	0.358	0.199
	gender × fresh milk	0.503	0.351	0.152
	gender × non-dairy creamer	−0.465	0.431	0.280
	gender × subsidiary of famous tea brand	−0.538	0.353	0.128
	gender × new brand	0.033	0.311	0.916
	education × provided by famous tea enterprises	−0.496	0.229	0.030
	education × black tea	0.068	0.345	0.844
	education × green tea	0.508	0.402	0.206
	education × jasmine tea	0.091	0.352	0.797
	education × fresh milk	−0.464	0.286	0.104
	education × non-dairy creamer	−0.404	0.343	0.238
	education × subsidiary of famous tea brand	0.583	0.343	0.090
	education × new brand	0.225	0.236	0.341
	education × ASC	−0.723	0.598	0.227
	frequency × ASC	−1.083	0.426	0.011
	frequency × provided by famous tea enterprises	0.011	0.139	0.938
	frequency × black tea	0.211	0.241	0.379
	frequency × green tea	0.134	0.267	0.616
	frequency × jasmine tea	0.056	0.211	0.792
	frequency × fresh milk	0.196	0.208	0.347
	frequency × non-dairy creamer	0.228	0.242	0.346
	frequency × subsidiary of famous tea brand	0.011	0.198	0.955
	frequency × new brand	−0.187	0.180	0.299
	allowance × ASC	−0.491	0.303	0.105
	allowance × provided by famous tea enterprises	0.228	0.124	0.065
	allowance × black tea	−0.368	0.184	0.045
	allowance × green tea	−0.449	0.219	0.040
	allowance × jasmine tea	−0.175	0.179	0.326
	allowance × fresh milk	0.026	0.168	0.875
	allowance × non-dairy creamer	−0.377	0.197	0.055
	allowance × subsidiary of famous tea brand	−0.115	0.163	0.482
	allowance × new brand	−0.074	0.142	0.601
Random parameter distributions	ASC	4.431	0.463	0.000
	Provided by famous tea enterprises	0.239	0.385	0.535
	Black tea	1.256	0.334	0.000
	Green tea	0.031	0.538	0.954
	Jasmine tea	−0.027	0.481	0.955
	Fresh milk	1.661	0.288	0.000
	Non-dairy creamer	2.229	0.371	0.000
	Subsidiary of famous tea brand	0.672	0.368	0.068
	New brand	−0.477	0.487	0.327
Log-likelihood		−1411.738		
LR chi2 (7)		657.910		

Note: “frequency” indicates frequency of weekly drinking milk tea; “allowance” indicates monthly allowance.

**Table 5:** WTP for different levels of attributes.

Attributes	RPL model	
	Mean (CNY)	CI
		[5%, 95%]
Provided by famous tea enterprises	1.12	[0.09, 2.16]
Black tea	0.44	[−1.26, 2.15]
Green tea	−0.26	[−2.09, 1.57]
Jasmine tea	0.41	[−1.05, 1.88]
Fresh milk	0.47	[−0.94, 1.88]
Non-dairy creamer	−10.40	[−14.08, −6.72]
Subsidiary of famous tea brand	1.08	[−0.32, 2.49]
New brand	−1.43	[−2.81, −0.05]

## Data Availability

Data are contained within the article.
